# Effect of ellagic acid on BDNF/PI3K/AKT-mediated signaling pathways in mouse models of depression

**DOI:** 10.22038/ijbms.2025.81230.17580

**Published:** 2025

**Authors:** Hatice Aslı Bedel, Coşkun Usta

**Affiliations:** 1 Süleyman Demirel University Faculty of Pharmacy Department of Pharmacology, Türkiye; 2 Akdeniz University Faculty of Medicine Department of Medicinal Pharmacology, Türkiye

**Keywords:** AKT, BDNF, Depression, Ellagic acid, PI3K

## Abstract

**Objective(s):**

The aim of this study is to investigate the possible role of the hippocampal BDNF-PI3K-AKT signaling pathway in the antidepressant-like activity of ellagic acid (EA) in mice.

**Materials and Methods:**

Male BALB/C mice were divided into 5 groups; vehicle (0.1 ml/day), sertraline (5mg/kg), EA (1 mg/kg), EA+BKM120 (PI3K inhibitor), EA+MK2206 (AKT inhibitor). EA, sertraline and vehicle were injected intraperitoneally for 14 days. Locomotor activity was determined by open field test. The tail suspension test was used to detect the antidepressant-like effect. After behavioral tests, hippocampal tissue was obtained and Western blot analyzes were performed for BDNF and pAKT1.

**Results:**

Sertraline and EA provided a reduction in immobility time in the tail suspension test when compared with the control group. BKM120 and MK2206 administration reversed this effect of EA. No statistical difference was found between groups in terms of locomotor activity. EA treatment caused an increase in hippocampal BDNF and pAKT1 levels in mice. While inhibitory agent administrations did not affect the increase of BDNF induced by EA, MK2206 administration reversed the increase in pAKT1 observed with EA.

**Conclusion:**

It has shown that EA has an antidepressant-like effect in mice without changing locomotor activity, and this effect may be mediated by the BDNF-PI3K-AKT signaling pathway.

## Introduction

Major depressive disorder (MDD) is a multifactorial psychiatric disorder that can be caused by genetic, social, and biochemical factors. MDD causes a burden. To eliminate this burden, procedures such as pharmacotherapy, psychotherapy, electroconvulsive therapy (ECT), deep brain stimulation, and lifestyle exercises are being used. Antidepressant medications are widely used to treat mood disorders. Antidepressants may cause various side effects (QT prolongation, gastrointestinal toxicity, anticholinergic effects, weight gain, sexual dysfunction, orthostatic hypotension, insomnia/agitation, drowsiness, etc.) (1). In clinic, patient compliance is disrupted due to these side effects and delayed onset and treatment resistance (2, 3). Understanding the pathophysiology of MDD enables us to generate new drugs. In recent years, herbal drugs have been investigated for the treatment of MDD.

Neurotrophins are growth factors that regulate neuronal growth, survival, and differentiation in the nervous system (4). Nerve growth factor and brain-derived neurotrophic factor (BDNF) are the most important members of the neurotrophin family (5). When BDNF binds to its receptor TrkB, three different signaling pathways are activated: Ras-PI3K-Akt, Ras-MAP kinase-Erk, and phospholipase Cγ (6). The PI3K/AKT pathway plays a role in cell survival, proliferation, cytoskeletal restructuring, and secretion events (7).

A meta-analysis conducted in 2017 found that antidepressant treatment for up to 12 weeks increased serum/plasma BDNF levels. (8). A recent meta-analysis showed that ECT application increased blood BDNF levels and significantly reduced depressive symptoms (9). Therefore, antidepressant drugs may provide beneficial effects through their interaction with BDNF.

Ellagic acid (EA) is a symmetric polyphenol. EA is found in strawberry, raspberry, blackberry, blueberry, goji berry, pomegranate, grape, walnut, chestnut, and a type of edible mushroom (*Fistulina hepatica*) in free form or as a complex derivative of ellagitannins (10-12). Huang *et al.* showed that EA reversed depression-like phenotypes and hippocampal damage caused by chronic unpredictable mild stress, and EA increased BDNF and serotonin levels (13). Our previous study found that EA has an antidepressant-like effect mediated by hippocampal BDNF increase (14). In this study, we aimed to determine the role of the BDNF-PI3K-AKT pathway among the possible mechanisms underlying the antidepressant-like effect of EA.

## Materials and Methods

Male 2-3 month-old Albino mice (15-30 g) were used in the study. During the experiment, the animals were housed in their cages at a room temperature of 22-25 ^°^C and exposed to 12 hours of light and 12 hours of darkness. The animals were kept in cages at a room temperature of 22-25 ^°^C with a 12-hour light and 12-hour darkness cycle during the experiment.Animals were fed standard food and water. This study was carried out according to ethical rules by considering animal welfare. Akdeniz University Animal Experiments Local Ethics Committee approved the study protocol. (Protocol number:927/2019.07.04)

Experimental animals were divided into five groups (n=10 per group). Mice in the first group received 0.1 ml vehicle (10% DMSO); sertraline (5 mg/kg) was administered to the second group, and EA (1 mg/kg) was administered to the third group. All chemicals were given intraperitoneally once a day for 14 days. The fourth group (EA+BKM120) was given 14 days of EA, followed by BKM-120 (PI3K inhibitor) on the last day. In the fifth group (EA+MK2206), after 14 days of EA treatment, MK-2206 (AKT inhibitor) was given on the last day. After the last drug treatment (the 14th day), the animals were brought to the room where behavioral tests would be performed and waited for half an hour to acclimate to the room. Afterward, open field and tail suspension tests (TST) were applied. After the behavioral experiments, the animals were sacrificed, and their hippocampus was removed. Western blot analysis was performed to determine BDNF and AKT1 protein levels in the hippocampus. 

Open Field Test: A square area made of black acrylic material 40 cm long * 40 cm high* 40 cm wide was divided into 16 equal parts. The mouse was placed in the middle of the apparatus, and a camera recorded its movements over five minutes as it moved around and explored its environment. After each mouse, the area was cleaned with 70% ethanol. The total number of squares crossed was calculated (15).

Tail Suspension Test: During the test, the mouse was suspended from its tail, and the duration of inactivity was recorded by camera for six minutes. The first two minutes of the experiment were considered the acclimation period, and the time the experimental animal remained immobile in the last four minutes was recorded in seconds (16).

Western blot: Mouse hippocampus tissues were dissected. Proteins were separated using 10% SDS-PAGE and transferred to polyvinylidene fluoride membranes. The membranes were incubated for 2 h with 5% skimmed milk or 5% BSA at room temperature and overnight at 4˚C with primary antibodies against BDNF (Elabscience, E-AB-18244), GAPDH (Elabscience, E-AB-40337), pAKT1 (Thr308) (Elabscience, E-AB-21082) and AKT1 (Elabscience, E-AB-63467) The next morning, the membrane was washed three times with TBST for ten minutes each. A secondary antibody (Anti-rabbit, Elabscience, E-AB-1003) suitable for the primary antibody was prepared at a ratio of 1:5,000 and incubated for one hour in a shaker at room temperature. After washing with washing solution, membranes were incubated with ECL for four minutes at room temperature. The bands were visualized using the bioanalytical imaging system (Azure Biosystems Model: c280), and the necessary calculations were made using the ImageJ software program.

### Statistical analysis

The results were statistically analyzed using the Graph-Pad computer program, using one-way analysis of variance (One-way ANOVA) followed by Dunnett’s test. *P*-values below 0.05 were considered significant. All values are expressed as mean±standard error.

## Results

### Open field test results

**Figure 1:**
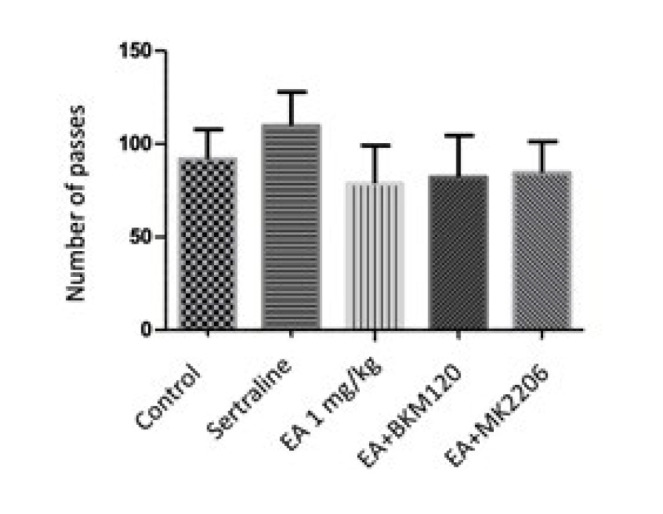
Effects of EA, EA+BKM120, EA+MK2206, and sertraline treatment in open field test in mice

The intervention groups (EA 1 mg/kg, sertraline, EA+BKM120, and EA+MK2206) showed no statistical difference in locomotor activity compared with the control group (Figure 1).

### Tail suspension test results

**Figure 2:**
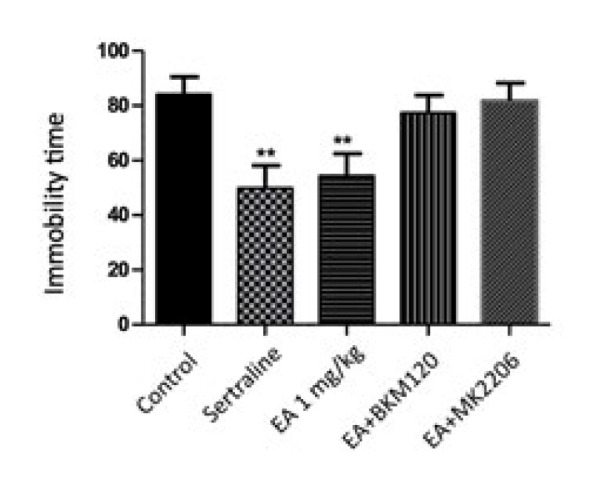
Effects of EA, EA+BKM120, EA+MK2206, and sertraline treatment on the tail suspension test in mice

It was observed that there was a statistically significant decrease in immobility time with EA 1 mg/kg and sertraline treatment compared to the control group (*P*<0.01). However, although it reduced the immobility time in the EA+BKM120 and EA+MK2206 groups compared to the control group, no statistically significant difference was detected (Figure 2).

### Western blot results for BDNF protein and AKT1 protein activation

**Figure 3:**
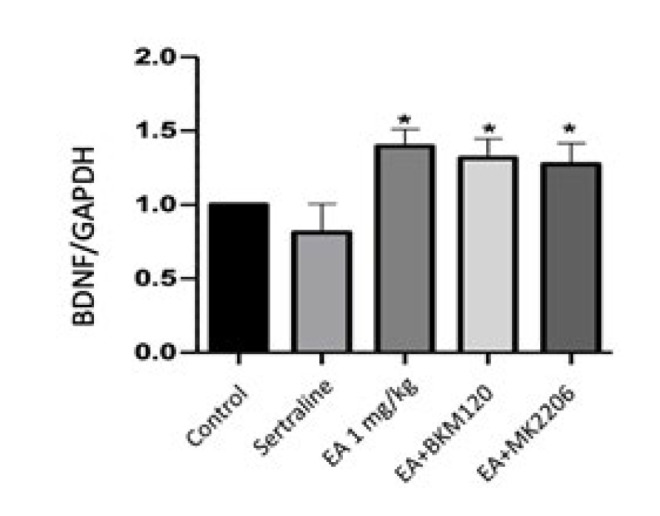
Effect of EA, EA+BKM120, EA+MK2206, and sertraline treatment on BDNF levels in mouse hippocampus

BDNF levels in the mouse hippocampus were proportioned to GAPDH, and the ratio of the control group was accepted as one. The other groups were normalized to the control group. There was no statistically significant decrease in BDNF levels in the sertraline group compared to the control group. EA 1 mg/kg, EA+BKM120, and EA+MK2206 groups increased BDNF levels statistically significantly compared to the control group (*P*<0.05) (Figure 3).

**Figure 4:**
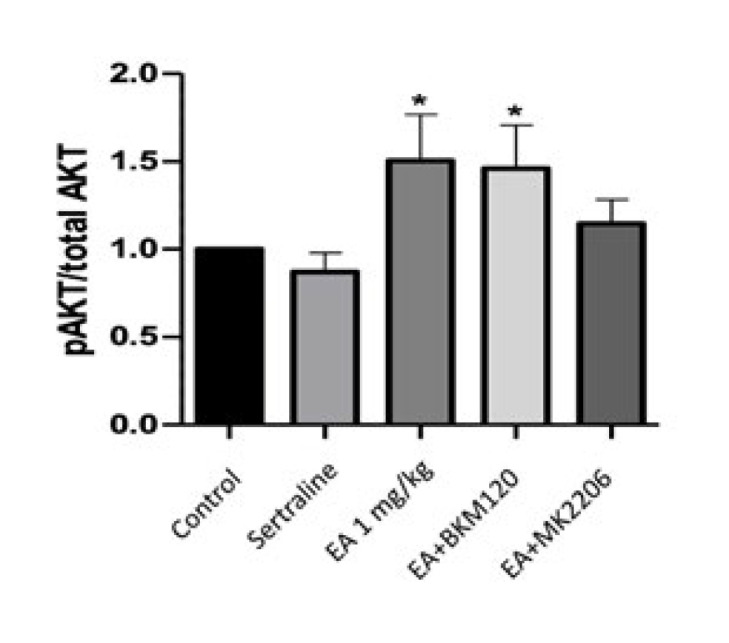
Effect of EA, EA+BKM120, EA+MK2206, and sertraline treatment on AKT1 phosphorylation in mouse hippocampus

pAKT1 levels in the mouse hippocampus were proportioned to total AKT1, the control group’s ratio was accepted as one, and the other groups were normalized to the control group. EA 1 mg/kg and EA+BKM120 groups increased AKT1 phosphorylation statistically significantly compared to the control group (*P*<0.05). No statistically significant difference was observed in AKT1 phosphorylation in the sertraline and EA+MK2206 group compared to the control group EA dramatically up-regulated levels of p-Akt, and this up-regulation was partially reversed in the presence of the Akt inhibitor MK-2206 (Figure 4).

## Discussion

To understand the multifactorial nature of depression, various hypotheses, such as the neuroplasticity hypothesis and the inflammation hypothesis, have been developed. According to the neuroplasticity hypothesis, BDNF levels decrease in depression, and this decrease is reversed with antidepressant treatment. The role of BDNF in the pathophysiology of depression has been intensively investigated for the last 20 years (17). Various studies have shown that bioactive molecules obtained from medicinal plants have antidepressant potential by acting through different pathways (18, 19). EA is a herbal molecule that is generally considered safe. Although there are bioavailability problems, the metabolites produced by the intestinal microbiota have also been shown to affect health positively. (20). EA is a plant secondary metabolite that has attracted attention in recent years with its antioxidant (21), anti-inflammatory (22), and anticarcinogenic (23) properties.

It has been demonstrated that herbal treatments may produce their antidepressant effects with BDNF-mediated responses. For example, liquiritigenin found in licorice root has shown antidepressant-like activity similar to fluoxetine in behavioral tests; this effect has been shown to be associated with BDNF/TrkB-mediated PI3K/AKT/GSK3 signaling in the hippocampus (24). Consumption of carotenoids, lycopene, and zeaxanthin has been associated with antidepressive symptoms with interaction of BDNF *in silico* (25).

In our study, the effects of EA were investigated by determining mouse hippocampal BDNF protein levels by western blot method, and it was shown that sertraline (5 mg/kg, 14 days) did not change the amount of BDNF in the mouse hippocampus. Consistent with our study, no statistically significant difference was detected in BDNF levels in the hippocampus of rats administered sertraline (5 mg/kg, 14 days) on the same day and dose, according to ELISA test results (26). In another study, fluoxetine, a selective serotonin reuptake inhibitor (SSRI), and sertraline were administered to mice for four weeks. Compared to the control group, BDNF levels in the hippocampus did not create a statistically significant change according to western blot results. However, chronic unpredictable mild stress reduced hippocampal BDNF levels compared to the control group, and this decrease was reversed when fluoxetine was given to the chronic stress group for four weeks (27). As can be understood from these studies, while SSRI group antidepressants do not affect BDNF levels in control group animals, they reverse the BDNF decrease caused by chronic stress.

One of the BDNF intracellular signaling pathways associated with the antidepressant-like effect is the PI3K/AKT pathway. An antidepressant-like effect was observed in the TST and forced swimming test in animals administered total saikosaponin, and it was shown that this effect was mediated by BDNF/pPI3K/pAKT up-regulation (28). In our previous study, we showed that EA has an antidepressant-like effect and increases BDNF levels in the mouse hippocampus. Moreover, in this study, we investigated whether the PI3K/AKT pathway plays a role in the effect of EA. We found that EA treatment reduced the immobility time in TST, and this effect was blocked in the presence of PI3K inhibitor (BKM120) or AKT inhibitor (MK2206). In a similar study, Pochwat *et al.* found that the immobility time in TST decreased after a single dose of hyperforin administration. The effects seen with hyperforin were blocked in the group given MK2206 (20 mg/kg) at the dose used in our study before hyperforin administration (29). Similarly, the antidepressant-like effects observed with vanillic acid were also eliminated with MK-2206 (60 mg/kg)(30).

In our study, EA increased hippocampal AKT1 phosphorylation. While BKM120 administration to animals receiving EA treatment did not change this increase, MK2206 administration disappeared EA’s increasing effect on AKT1 levels. Consistent with our study, in the study by Roy *et al.*, BKM120 administration did not change AKT^T308^ and AKT^S473^ phosphorylation in the hippocampus compared to the control group (31).

It has been shown that Huperzine A (the active ingredient found in the *Huperzia serrata* plant) corrects the oxidative glutamate toxicity produced in hippocampal HT22 cells, and the modulation of the BDNF/TrkB-dependent PI3K/AKT/mTOR signaling pathway is effective in this neuroprotective effect (32). In an animal study, rats were given oral EA at a dose of 50 mg/kg, and it was determined that hippocampal BDNF, pAKT, and pPI3K levels increased with chronic EA treatment compared to the control group (33). In our study, EA significantly increased BDNF and pAKT1 levels compared to the control group. Therefore, it may be assumed that this pathway may be used in the antidepressant-like effect of EA.

The mean difference is not statistically significant when compared with the control group, *P>*0.05; EA: Ellagic acid

**The mean difference is significant when compared with the control group, *P<*0.01; EA: Ellagic acid

*The mean difference is significant when compared with the control group, *P<*0.05; EA: Ellagic acid

*The mean difference is significant when compared with the control group, *P<*0.05; EA: Ellagic acid

## Conclusion

Very few studies exist about the possible antidepressant effectiveness of EA and its underlying mechanisms. Our study showed that EA has an antidepressant-like effect in mice without changing locomotor activity, and the BDNF-PI3K-AKT signaling pathway may mediate this effect. Recently, EA has been subject to clinical trials. Ellagic acid supplementation had a favorable effect on depression in MS patients (34) and also in MDD patients (35). EA seems a promising agent in depression. However, more preclinical and clinical studies are needed to evaluate whether the results obtained from our study can pave the way for clinical application. 

## Declaration

We have not used any AI tools or technologies to prepare this manuscript.
